# Enhanced antibacterial and anti-inflammatory properties of Hwangryeonhaedok-tang fermented with Kimchi-derived *Lactiplantibacillus plantarum* WiKim0111 for potential acne treatment

**DOI:** 10.1007/s42770-025-01703-z

**Published:** 2025-06-23

**Authors:** Ho Jae Lee, Chang Hee Jeong, Tae-Woon Kim, Sang Wan Seo, Do-Won Jeong, Sung Wook Hong

**Affiliations:** 1 Technology Innovation Research Division, World Institute of Kimchi, Gwangju, 61755 Republic of Korea; 2 Department of Biomedical Laboratory Science, Honam University, Gwangju, 62399 Republic of Korea; 3 Department of Food and Nutrition, Dongduk Women’s University, Seoul, 02748 Republic of Korea

**Keywords:** *Lactiplantibacillus plantarum*, Hwangryunhaedok-tang, Anti-acne effect, Anti-bacterial agents, Anti-inflammatory agents, Fermentation

## Abstract

Recent years have seen an increase in the incidence of acne in adults and teenagers resulting in active research in acne treatment. In the present study, we aimed to improve the anti-acne effects of Hwangryeonhaedok-tang (HHT) through fermentation using a kimchi-derived lactic acid bacterium. *Lactiplantibacillus plantarum* WiKim0111 (*Lpb. plantarum* WiKim0111) was selected to ferment HHT due to its strong antioxidant activity and its ability to effectively survive and grow in the HHT medium. The antimicrobial activity of HHT and fermented HHT (FHHT) against *Cutibacterium acnes* (*C. acnes*) was determined using an agar well diffusion assay. FHHT exhibited stronger antimicrobial, radical scavenging, and superoxide dismutase activity compared to HHT. RAW 264.7 cells pretreated HHT or FHHT showed significantly lower production of nitric oxide, interleukin (IL) -1β, and IL-6 than lipopolysaccharide -treated cells, with FHHT demonstrating higher efficiency than HHT. Overall, HHT fermented by *Lpb. plantarum* WiKim0111 displayed enhanced antioxidant and anti-inflammatory properties, as well as greater antibacterial activity against *C. acnes* compared to unfermented HHT. These findings suggest that FHHT may help mitigate acne symptoms by reducing oxidative stress, modulating inflammation, and directly inhibiting the growth of *C. acnes*. Further in vitro studies focusing on sebum-producing skin cells, as well as clinical trials evaluating the topical or oral application of FHHT, would be valuable to confirm its efficacy and safety in acne treatment.

## Introduction

Acne is a common epidermal inflammatory condition of the pilosebaceous unit. While the prevalence of acne is considerably higher in teenagers, recent trends indicate increasing incidence in adults as well. This may be attributed to dietary factors, including the increased intake of foods with high glycemic index, saturated fat, and trans fat [1]. Sebum hypersecretion by androgenic hormones, follicular hyperkeratinization, *Cutibacterium acnes* (formerly *Propionibacterium acnes*) proliferation, and the resultant inflammation have been reported as causative factors for acne [2].

*C. acnes* is a Gram-positive anaerobic bacterium that resides on the skin as a commensal microorganism [3]. Predominantly found at sebaceous sites, it plays a key part in regulating skin homeostasis and preventing pathogen colonization [4]. However, *C. acnes* is also a frequent cause of opportunistic skin infections and acne [5]. Previous studies have shown that *C. acnes* promotes keratinocyte migration and proliferation due to oxidative stress and inflammatory responses, potentially resulting in acne [6].

Hwangryeonhaedok-tang (HHT) has been widely employed in oriental medicine for treating skin diseases such as atopic dermatitis [7] and seborrheic dermatitis [8]. HHT primarily contains Phellodendri cortex (PC), Scutellaria baicalensis (SB), Coptis chinensis (CC), and Gardenia jasminoides Ellis (GJE). Of these, PC and CC are known known for their anti-inflammatory and anti-hyperglycemic activities due to the presence of alkaloids like berberine and palmatine [9]. The antibacterial activity of HHT against *Staphylococcus epidermidis* and *Staphylococcus aureus* has been observed at high concentrations [10]. Therefore, additional studies are required to improve the efficacy of HHT at lower concentrations.

Fermentation by microorganisms to enhance the effect of natural ingredients has been widely studied and applied in various fields, ranging from pharmaceuticals and cosmetics to food and beverages [11]. Various organisms including bacteria, yeast, and mold, particularly lactic acid bacteria (LAB), are commonly employed in the fermentation process. Recent studies have highlighted the health-promoting effects of LAB derived from kimchi, including antioxidant, anti-inflammatory, and probiotic activities [12]. Previously, Mun et al. showed that fermentation of *Sargassum thunbergii* using *Lactobacillus sp. SH-1* isolated from watery kimchi increases the anti-inflammatory activity of *S. thunbergii* by inhibiting pro-inflammatory cytokine expression in macrophage cells [13].

Therefore, in this study, we evaluated the antibacterial, antioxidant, and anti-inflammatory activities of HHT fermented with kimchi-derived *Lactiplantibacillus plantarum* WiKim0111 (*Lpb. plantarum* WiKim0111), and compared them with those of unfermented HHT to investigate the enhanced functional potential of fermentation for acne-related applications.

## Materials and methods

### Isolation-cultivation of strains from Kimchi

LAB were isolated from a variety of kimchi such as cabbage kimchi, young radish kimchi, cubed radish kimchi, and radish water kimchi. Each 10 g of kimchi sample was put down in a stomacher bag in 90 mL of 0.85% (w/v) sodium chloride and stomached for 5 min (Seward Laboratory Systems Inc., Bohemia, NY, USA). The diluted solution (0.1 mL) was serially diluted 10-fold and spread onto the surface of De Man, Rogosa, and Sharpe (MRS) agar medium (DifcoTM, BD Biosciences, Sparks, MD, USA). All plates were cultured at 30 °C for 48 h. Isolates from kimchi were cultured in a broth prepared by hot water extraction of a mixture of PC, SB, CC, and GJE. For comparison, *Lacticaseibacillus rhamnosus* GG (*Lcb*. *rhamnosus* GG) and *Lpb. plantarum* WCFS1 strains were also cultured under the same conditions. Bacterial growth was assessed every 12 h over a 36-hour period by plating on MRS agar and monitoring colony formation

### HHT and FHHT Preparation

CC, PC, GJE, and SB were obtained from an herbal medicine wholesaler (Gwangju, Korea). HHT was prepared as follows: 20 g of each ingredient and 5 g of yeast extract was added to 1 L of deionized water (DW), boiled at 121 °C for 2 h, filtered through a cotton filter cloth, and finally centrifuged at 8,500 × g for 15 min to collect the supernatant. Fermented HHT (FHHT) was prepared by inoculating the selected strain (1.5 × 10^8^ CFU/mL), which was cultured overnight the day before, into HHT and incubating it at 37 °C for 48 h. HHT and FHHT supernatants were obtained through centrifugation (8,000 × g, 10 min) and filtration using a membrane filter (0.45 μm, Advantec Co., Japan). HHT and FHHT supernatant were freeze-dried and then stored at -80 °C. In addition, since *Lpb. plantarum*, a LAB, produces acidic end products during fermentation, the pH of HHT decreased from around 6.35 pre-fermentation to 4.52 post-fermentation. A low pH promotes an unfavorable environment for the growth of other microorganisms including pathogenic bacteria [14]. Therefore, the low pH of FHHT may affect the experimental results, and thus was adjusted using NaOH to match that of HHT (approximately pH = 6.30) before being used in experiments.

### Measurement of organic acid content

Citric acid, malic acid, lactic acid, and acetic acid were quantified using a modified high-performance liquid chromatography (HPLC) method [15]. Initially, 2.5 g of HHT or FHHT were placed in a 50 mL conical tube (SPL Life Science, Gyeonggi-do, Korea) and diluted to a final weight of 30 g with distilled water. The mixture was then heated to 85 °C for 25 min and centrifuged at 3,000 rpm for 10 min (Eppendorf, Hamburg, Germany). The resulting supernatant was filtered through a 0.45-µm membrane filter (Advantec MFS, Japan) before being injected into the HPLC system. The analysis was performed using a Dionex Ultimate 3000 system equipped with a RefractoMax521 RI detector (Thermo Fisher Scientific, Waltham, MA, USA). The HPLC conditions were as follows: wavelength of 210 nm, mobile phase of 0.01 N H_2_SO_4_, separation column Aminex HPX-87 H (300 × 7.8 mm; Bio-Rad, Hercules, CA, USA), flow rate of 0.5 mL/min, injection volume of 10 µL, and oven temperature set to 40 °C.

### Measurement of SOD activity

The superoxide dismutase (SOD) assay was conducted by using the OxiTec™ SOD assay kit (Biomax, Seoul, Korea) following a manufacturer’s instructions. The HHT or FHHT supernatant (200 µL) was mixed with 2.6 mL of Tris·HCl buffer (50 mM tris (hydroxymethyl) aminomethane, 10 mM EDTA, pH 8.5) and 200 µL of 7.2 mM pylogallol before incubation at room temperature (RT; 23–26 °C) for 10 min. In order to stop the reaction, 1 N HCl was added and the absorbance was determined at 450 nm by a microplate reader (Infinite 200 pro, Tecan Austria GmbH, Grödig, Austria) [16].

$$\text{SOD-like activity} (\%) = [1-(\text{ODsample/ODblank})\times100]$$

### Identification of selected strain

The selected strain was identified by a DNeasy tissue kit (Qiagen, Valencia, CA, USA) following a manufacturer’s directions and previously described method [17]. The conserved region of the 16 S ribosomal RNA (rRNA) gene was amplified with the universal primer pairs (RP: (1492R) 5ʹ-GGTTACCTTGTTACGACTT-3ʹ and FP: (27 F) 5ʹ-AGAGTTTGATCCTGGCTCAG-3ʹ) and Takara Perfect Premix (Takara, Japan) using an Applied Biosystems thermal cycler (Thermo Fisher Scientific, Pittsburgh, PA, USA). The conditions of polymerase chain reaction (PCR) were as follows: a denaturation at 94 °C for 5 min; 30 cycles of denaturation at 94 °C for 45 s, annealing at 52 °C for 45 s, and extension at 72 °C for 1 min, and final elongation at 72 °C for 5 min. The 16 S rRNA was sequenced by a Genetic Analyzer (ABI 377, Applied Biosystems, Foster City, CA, USA). The sequenced 16 S rRNA gene was identified in the EZBioCloud database (www.ezbiocloud.net/eztaxon, accessed on October 13, 2021). A phylogenetic tree was built by neighbor-joining and maximum-parsimony criteria with bootstrap values inferred with 1,000 replications.

### Agar well diffusion assay

The antibacterial activity of HHT and FHHT was estimated by agar well diffusion assay following a previously described method [18]. *C. acnes* KACC 11,946 (1 × 10^5^ CFU/mL) cultured in Reinforced Clostridial Medium (RCM) was poured into a RCM plate in which 8 mm diameter well were made with a sterilized cork borer. One hundred microliters of each sample (DW, HHT, FHHT, HHT fermented with *Lacticaseibacillus rhamnosus* GG (LGG), and HHT fermented with *Lpb. plantarum* WCFS1 (WCFS1) at 400 µg/mL) were then added to each well. After incubation at 30 °C for 24 h, the clear zone was calculated in millimeters.

### Radical scavenging assays

The 2,2’-azino-bis(3-ethylbenzothiazoline-6-sulphonic acid) (ABTS; Sigma-Aldrich, St. Louis, MO, USA) assay was performed as described [19]. Briefly, ABTS reagent (70 mM) was mixed with potassium persulfate (24.5 mM) and reacted in a dark at RT for 12 h. The ABTS^+^ stock solution was then mixed with distilled water (DW) to obtain the working solution with an absorbance of 0.700 ± 0.02 at 734 nm and 100 µL was incubated with 50 µL of either HHT or FHHT supernatant in a 96-well plate in dark at RT for 30 min. L-Ascorbic acid (0.5%) was employed as a positive control. ABTS scavenging activity was determined by measuring the absorbance at 734 nm by a microplate reader.

The 2,2-diphenyl-1-picryl-hydrazyl (DPPH; Sigma-Aldrich) assay was conducted according to previous study [20]. Briefly, 100 µL of the DPPH solution was mixed with either 100 µL of HHT or FHHT supernatant in a 96-well plate and incubated in the dark at RT for 30 min. L-Ascorbic acid (0.5%) was employed as a positive control. DPPH scavenging activity was determined by measuring the absorbance at 517 nm by a microplate reader.

$$\begin{aligned}&\rm{Radical\, scavenging\, activity} (\%) \cr&\quad= [1-(\rm{ODsample/ODcontrol})\times100]\end{aligned}$$

### Cell culture method

Murine macrophage cells RAW264.7 (Korean Cell Line Bank, Jongno, Seoul, Korea) were cultured in Dulbecco’s modified Eagle’s medium (DMEM; Welgene Inc., Gyeongsan, Korea) supplemented with 10% fetal bovine serum and 1% penicillin/streptomycin (Welgene Inc.) at 37 °C under humidified conditions and 5% CO_2_, following a previously described method [20].

### Cell viability assay

To determine the cell viability of RAW 264.7, the 3-(4,5-dimethylthiazol-2-yl)-5-(3-carboxymethoxyphenyl)-2-(4-sulfophenyl)-2 H-tetrazolium) (MTS; Abcam; Cambridge, MA, USA) assay was conducted [21]. RAW264.7 cells were seeded at a density of 1 × 10⁵ cells/well in 96-well plates for the cell viability assay. The cells were cultured in a 96-well plate and exposed to HHT (25, 50, 100, 200, or 400 µg/mL), FHHT (25, 50, 100, 200, or 400 µg/mL), or sterile DW (control) for 24 h. Following exposure, the cells were further incubated for 3 h in fresh DMEM containing 10% MTS solution. The OD was determined at 490 nm by a microplate reader.

$$\rm{Cell\,viability} (\%) = (\rm{ODsample/ODcontrol})\times100$$

### NO production assay

The nitric oxide (NO) production level was determined using the Griess reaction. Briefly, 1 × 10^5^ cells/mL of RAW264.7 cells were seeded onto 6-well plates and grown to approximately 80% confluence. After pretreatment with HHT or FHHT supernatant (25, 50, 100, 200, and 400 µg/mL) for 1 h, the cells were challenged with lipopolysaccharide (LPS; 1 µg/mL) or phosphate-buffered saline (PBS; control) for 24 h. The supernatants were obtained via centrifugation at 3,800 × g for 15 min and mixed with Griess reagent. After incubation at room temperature (RT) for 15 min, the absorbance was determined at 540 nm by a microplate reader [22].

### Measurement of cytokine production

The levels of the pro-inflammatory cytokines IL-1β, IL-6, and TNF-α were determined in RAW264.7 cells using the Quantikine^®^ ELISA kit (R&D Systems, Minnesota, USA). Cells were seeded in serum-free medium to avoid interference from FBS-derived cytokines and incubated overnight. Four treatments were applied: PBS only (no LPS, no HHT/FHHT), LPS alone (1 µg/mL), HHT (25, 50, 100, 200, 400 µg/mL) with LPS, and FHHT (25, 50, 100, 200, 400 µg/mL) with LPS. For the HHT and FHHT treatments, cells were pretreated for 1 h before LPS (1 µg/mL) was added. After 24 h, the supernatants were collected by centrifugation at 3,800 × g for 15 min, and 100 µL of each sample was mixed with 100 µL of assay diluent in a 96-well plate. The plate was incubated at RT for 2 h and then aspirated, followed by the addition of 200 µL of conjugate to each well and a further 2 h of incubation at RT. After washing, an equal volume of substrate solution was added and incubated at RT for 2 h. Finally, 50 µL of stop solution was added, and the optical density was measured at 450 nm using a microplate reader [23].

### Statistical analysis

Data are shown as the mean ± standard deviation (SD; *n* = 3). Statistical significance was estimated using one-way analysis of variance (ANOVA) and Student’s t-test. The tests were carried out using SPSS–PASW statistics software version 19 (Chicago, IL, USA). Statistical significance was set at *p* < 0.05.

## Results and discussion

### Selection and identification of Kimchi lactic acid bacteria

**Fig. 1:**
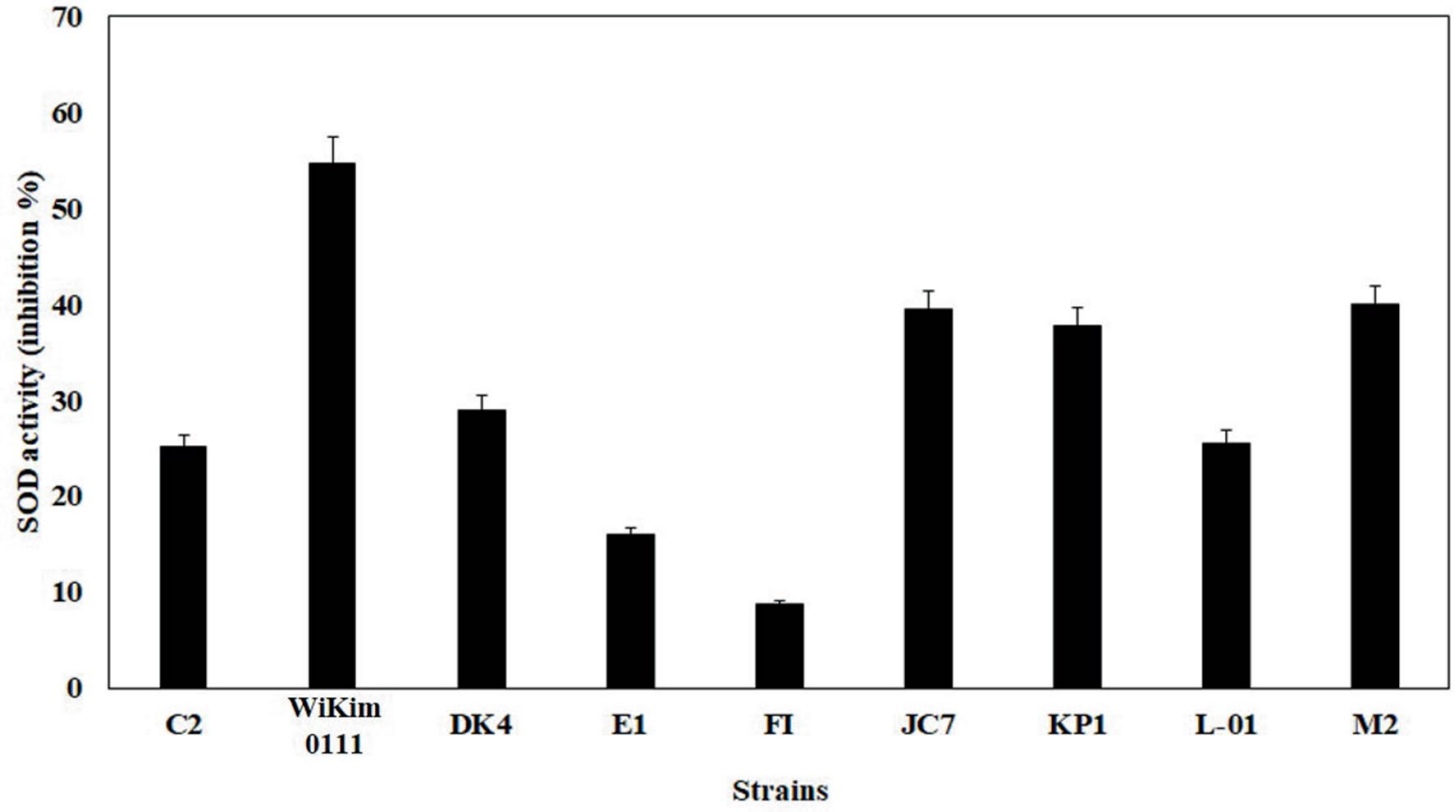
SOD activity of kimchi derived bacterial isolate supernatants. The SOD activity of the supernatants of selected strains was estimated using a microplate reader by measuring absorbance at 450 nm. Each strain was identified as follows: C2, *Lactiplantibacillus plantarum*; DK4, *Lpb. plantarum*; E1, *Lactobacillus pentosus*; FI, *L. pentosus*; JC7, *L. brevis*; KP1, *L. brevis*; L-01, *L. sakei*; M2, *Leuconostoc mesenteroides*. Note: SOD, superoxide dismutase

**Fig. 2:**
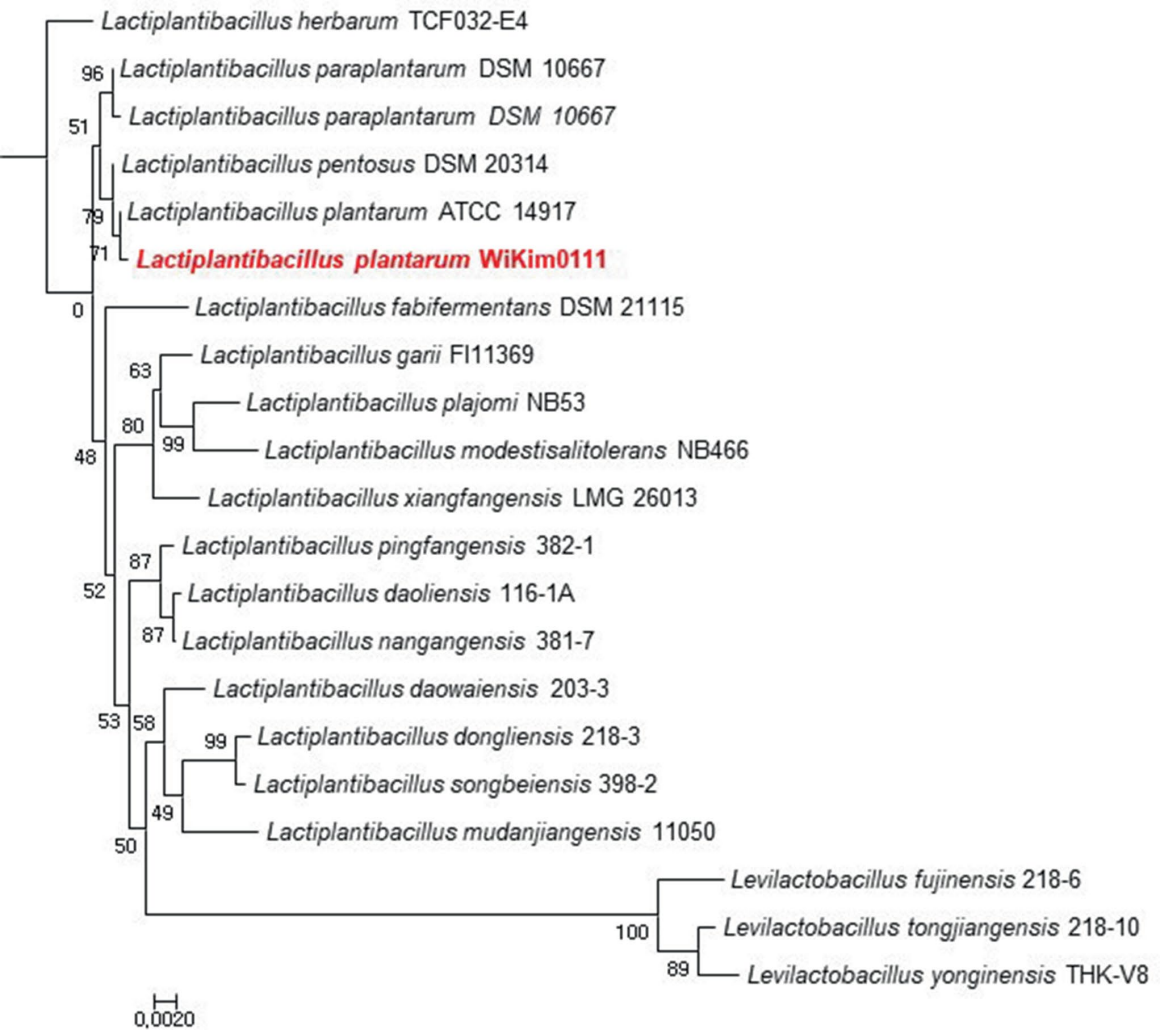
Phylogenetic analysis of *Lpb. plantarum* Wikim0111 based on 16 S rRNA sequence homology. Phylogenetic tree of *Lpb. plantarum* Wikim0111 was established using the neighbor-joining method. Bootstrap percentages for 1000 re-sampling are given. Note: *Lpb. plantarum* Wikim0111, *Lactiplantibacillus plantarum* Wikim0111

Among the 300 species investigated, 15 strains with acceptable growth in water extracts of the major ingredients of HHT were selected (data not shown), and of which Wikim0111 showed the strongest SOD activity (Fig. 1). To identify the Wikim0111 strain, phylogenetic relationships were investigated based on 16 S rRNA sequences, which were analyzed using the EzBioCloud database. Sequence analysis revealed Wikim0111 had a 16 S rRNA sequence that was > 99% similar to that of *Lactiplantibacillus plantarum* ATCC 14,917 (Fig. 2). The selected strain was identified as *Lpb. plantarum* and named *Lpb. plantarum* Wikim0111.

### Growth of Lpb. Plantarum WiKim0111 and probiotic strains in HHT

To evaluate the suitability of *Lpb. plantarum* WiKim0111 as an HHT-fermenting strain, viable cell counts were measured after inoculating *Lpb. plantarum* WiKim0111 (10⁸ CFU/mL) into a hot water extract prepared from a mixture of PC, SB, CC, and GJE (referred to as HHT). For comparison, *Lcb*. *rhamnosus* GG and *Lpb. plantarum* WCFS1 were also inoculated under the same conditions (Table 1). Viable cell numbers of *Lpb. plantarum* WiKim0111 increased continuously during fermentation and reached the highest level at 36 h (12.25 ± 0.11 Log CFU/mL), which was higher than those of *Lcb*. *rhamnosus* GG (11.63 ± 0.12 Log CFU/mL) and *Lpb. plantarum* WCFS1 (11.77 ± 0.09 Log CFU/mL). All strains showed viable cell counts exceeding 10^9^ CFU/mL by 36 h. Furthermore, inoculation of *Lpb. plantarum* WiKim0111 at an initial concentration of 10^8^ CFU/mL into HHT resulted in a final viable cell number of 2.5 × 10^12^ CFU/mL. Our results indicate that *Lpb. plantarum* Wikim0111 can be successfully used to ferment HHT. Several plant extracts are known to have antibacterial effects [24, 25] and in concordance with our findings, it has been demonstrated that LAB grow well in plant extracts. As previously reported, *Lactobacillus delbrueckii* subsp. *bulgaricus* and *Lactobacillus acidophilus* grew well in 1, 2, and 5% aloe extract [26], and *Lpb. plantarum* grows well in licorice extract [27]. While the mechanisms responsible for the enhanced growth of LAB in plant extracts are not well elucidated, a possible explanation is that plant polyphenols metabolized by LAB provide a favorable environment by modulating oxidative stress [28].

**Table 1:** Measurement of lactic acid bacteria growth in HTT

Ingredients	0 h (Log CFU/mL)	12 h (Log CFU/mL)	24 h (Log CFU/mL)	36 h (Log CFU/mL)
WiKim0111	8.42 ± 0.18	9.44 ± 0.09	11.42 ± 0.08	12.25 ± 0.11
LGG	8.63 ± 0.11	9.43 ± 0.08	10.44 ± 0.11	11.63 ± 0.12
WCFS1	8.18 ± 0.13	9.02 ± 0.13	9.94 ± 0.22	11.77 ± 0.09

*Note*: WiKim0111 HHT fermented *with Lactiplantibacillus plantarum* WiKim0111; LGG, HHT fermented with *Lacticaseibacillus rhamnosus* GG; WCFS1, HHT fermented with *Lactiplantibacillus plantarum* WCFS1;

### Organic acid content of HHT and FHHT

The content of organic acids, including citric acid, malic acid, lactic acid, and acetic acid, in HHT and FHHT was measured using HPLC analysis throughout the fermentation period (Table 2). Initially, only citric acid and malic acid were detected in HHT before fermentation. However, both acids were consumed during fermentation, and lactic acid and acetic acid were detected instead. Bensmira and Jiang noted that certain lactic acid bacteria (LAB) can produce acetoin and diacetyl using citric acid as a substrate [29]. Specifically, *L. plantarum* converts malic acid to lactic acid through the malolactic fermentation process [30, 31]. Compared to the initial values in HHT, the levels of lactic acid (9,129.87 mg/L) and acetic acid (723.2 mg/L) increased during fermentation. Lactic acid is a major end metabolite produced by the homofermentative metabolism of LAB [32], while acetic acid is a key metabolite in the heterofermentative metabolism of LAB [33]. Thus, our findings indicate that *L. plantarum* Wikim0111 can serve as an effective starter for HHT fermentation.

**Table 2:** Concentration of organic acid of HHT and FHHT

Organic acid	HHT	FHHT
Citric acid (mg/L)	1200.08	458.82
Malic acid (mg/L)	3510.65	2394.79
Lactic acid (mg/L)	N.D.	9129.87
Acetic acid (mg/L)	N.D.	723.20

*Note*: HHT, Hwangryeonhaedok-tang; FHHT, fermented HHT; N.D., not detected

### Antibacterial activity of HHT and FHHT

**Fig. 3:**
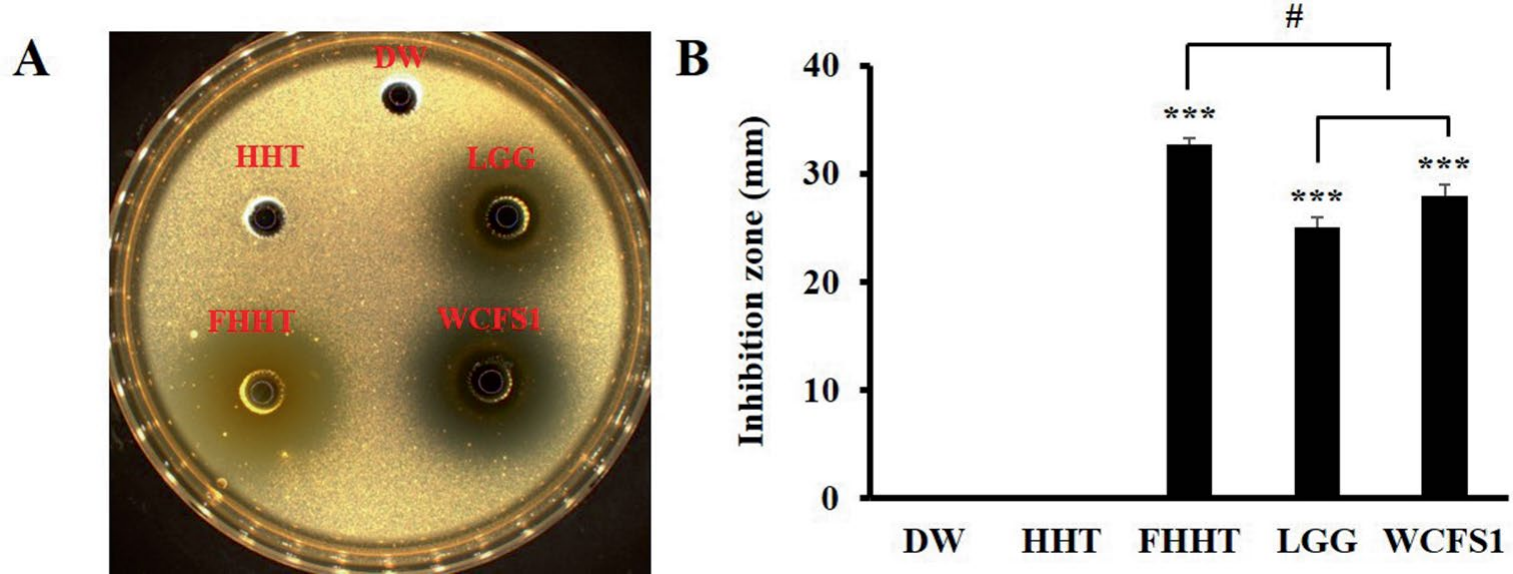
Antibacterial activity of HHT and its fermented forms against *Cutibacterium acnes* KACC 11,946. (**A**) Representative image showing the inhibition zone. (**B**) Quantification (mm) of the inhibition zone under DW, HHT, FHHT, LGG, and WCFS1 (mm). The results are represented as the mean ± SD based on four independent experiments. *** *p* < 0.001 indicates a significant difference w.r.t the control (DW). # *p* < 0.05 indicates a significant difference compared with FHHT. Note: HHT, Hwangryeonhaedok-tang; FHHT, HHT fermented with *Lactiplantibacillus plantarum* WiKim0111; LGG, HHT fermented with *Lacticaseibacillus rhamnosus* GG; WCFS1, HHT fermented with *Lactiplantibacillus plantarum* WCFS1; DW, deionized water

An agar well diffusion assay was performed to evaluate the antibacterial activity of HHT fermented with *Lpb. plantarum* WiKim0111 (FHHT) against *C. acnes* KACC 11,946 (Fig. 3). DW served as the negative control, while HHT fermented with *Lacticaseibacillus rhamnosus* GG (LGG) and HHT fermented with *Lpb. plantarum* WCFS1 were included as positive controls. Both LGG and *Lpb. plantarum* WCFS1 are well-characterized probiotic strains known to produce an array of antimicrobial metabolites, such as organic acids and bacteriocins, making them suitable reference strains for evaluating antibacterial efficacy [34, 35].

Among the tested samples, FHHT produced the largest inhibition zone, indicating a stronger antibacterial effect against *C. acnes* than HHT and the other fermented preparations (LGG and WCFS1). In contrast, the negative control (DW) did not exhibit any inhibition zone. These findings are consistent with the known ability of lactic acid bacteria (LAB) to synthesize inhibitory substances, including lactic acid, acetic acid, and bacteriocins, which can suppress pathogenic bacteria [36, 37]. In agreement with our results, a previous study demonstrated that Aloe supernatant fermented by *Lpb. plantarum* HM218749.1 showed antibacterial effects against *C. acnes* [38].

Taken together, these observations suggest that *Lpb. plantarum* WiKim0111 is able to ferment HHT efficiently, yielding metabolites that enhance its antibacterial activity against *C. acnes*. However, for skin application, adjusting the pH of FHHT to around 5.5—the typical skin surface pH—may be necessary to avoid potential side effects such as cutaneous dysbiosis [39, 40]. Additional in vivo research and clinical evaluations will be required to confirm the potential of FHHT as a complementary strategy for acne prevention and treatment.

### Antioxidant effect of HHT and FHHT

**Fig. 4:**
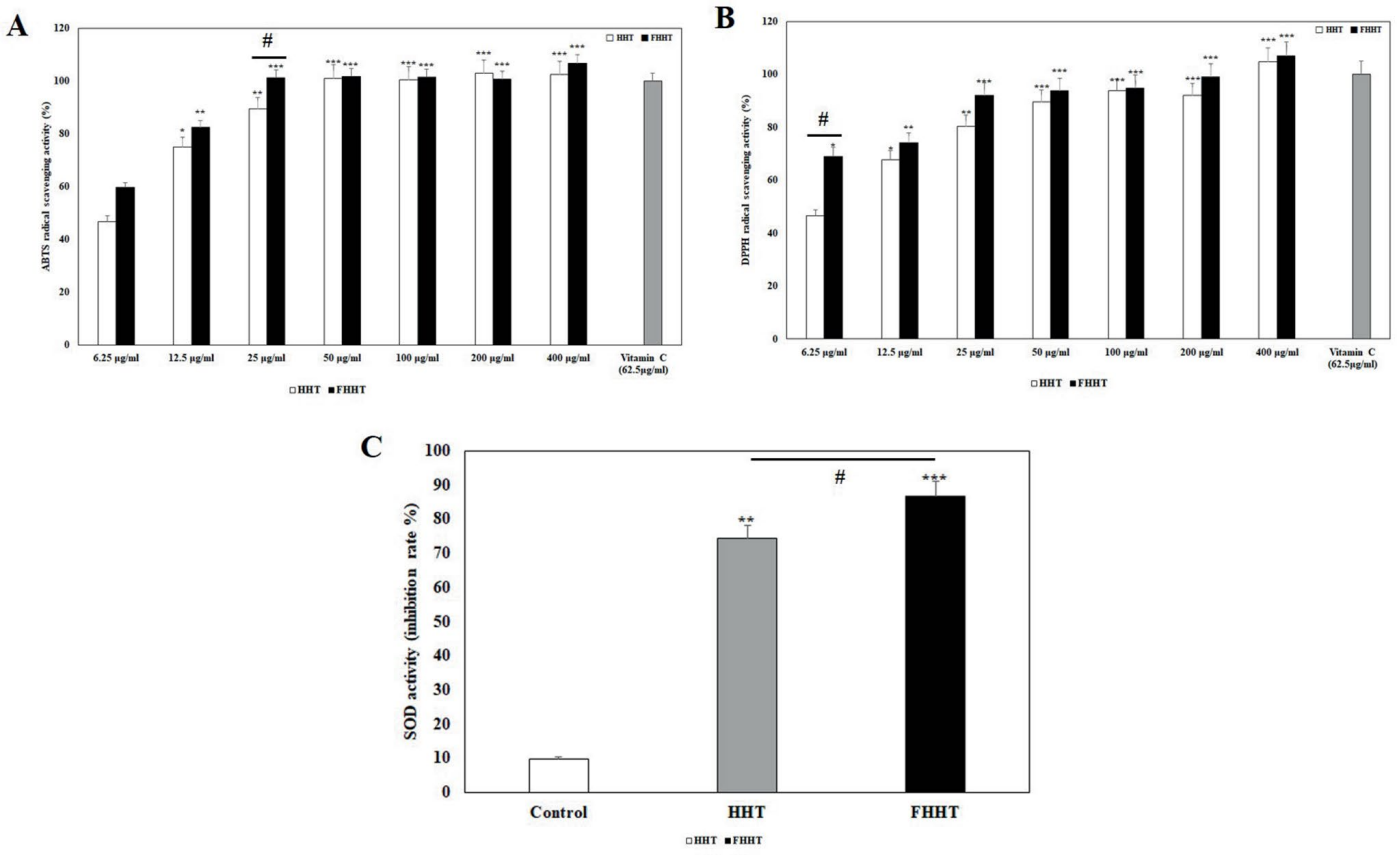
Antioxidant activity of HHT and FHHT. The antioxidant activity was determined using the (**A**) ABTS, (**B**) DPPH, and (**C**) SOD assays. The results are represented as mean ± SD from three independent experiments. * *p* < 0.05, ** *p* < 0.01, *** *p* < 0.001 indicates a significant difference w.r.t the control (DW). # *p* < 0.05 indicates a significant difference on HHT vs. FHHT. Note: HHT, Hwangryeonhaedok-tang; FHHT, fermented Hwangryeonhaedok-tang; ABTS, 2,2’-azino-bis(3-ethylbenzothiazoline-6-sulphonic acid); DPPH, 2,2-diphenyl-1-picryl-hydrazyl; SOD, superoxide dismutase; SD, standard deviation; DW, deionized water

Antioxidants play an important role in alleviating skin inflammatory responses (e.g., acne) since *C. acnes* is reported to induce oxidative stress in keratinocytes, leading to skin inflammation [41]. We investigated the antioxidant activity of HHT and FHHT by performing ABTS and DPPH radical scavenging and SOD assays. While both HHT and FHHT showed significantly higher ABTS radical scavenging activity than the vehicle control (Fig. 4A), FHHT had a markedly higher ABTS radical scavenging activity than that of HHT at concentrations of 25 µg/mL or less. In the DPPH test, FHHT exhibited significantly higher radical scavenging activity than the vehicle control even at a low concentration of 6.25 µg/mL (Fig. 4B) and HHT at all concentrations tested. Additionally, FHHT and HHT exhibited 86.64% and 74.39% SOD activity, respectively, at a concentration of 25 µg/mL (Fig. 4C). Collectively these results establish that FHHT exhibits higher antioxidant activity than that of HHT, even at concentrations below 25 mg/mL. Extracts of medicinal plants such as SB, PC, and GJE are known to contain abundant phenolic compounds [42], high concentrations of which negatively impact bacteria by inducing cell wall and membrane damage, and pH gradient dissipation [43]. Although we observed enhanced antioxidant activity in FHHT, the specific metabolites responsible for this improvement were not identified in the present study. Previous research indicates that phenolic compounds can be biotransformed by LAB into metabolites with higher biological activity, such as alkyl catechols, which are known to activate antioxidant-related pathways [44]. Based on these findings, we speculate that similar metabolic processes may have occurred during the fermentation of HHT by *Lpb. plantarum* WiKim0111. Further metabolite profiling would be necessary to directly confirm the involvement of specific phenolic derivatives in the observed antioxidant activity.

### Anti-inflammatory activity of HHT and FHHT

**Fig. 5:**
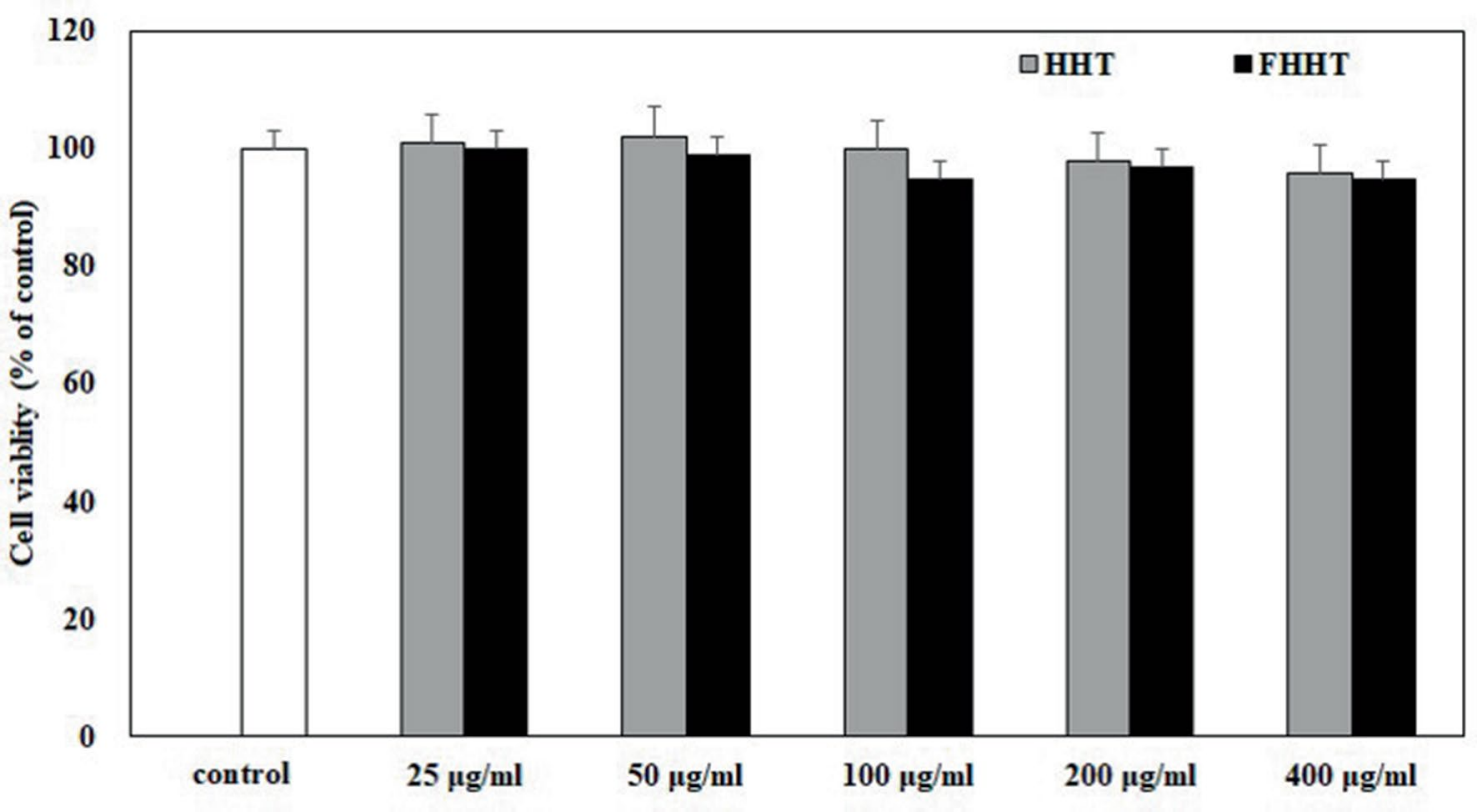
Effect of HHT and FHHT on the viability of RAW 264.7 cells. The results are represented as mean ± SD from three independent experiments. Note: MTS, 3-(4,5-dimethylthiazol-2-yl)-5-(3-carboxymethoxyphenyl)-2-(4-sulfophenyl)-2 H-tetrazolium); HHT, Hwangryeonhaedok-tang; FHHT, fermented Hwangryeonhaedok-tang; SD, standard deviation

**Fig. 6:**
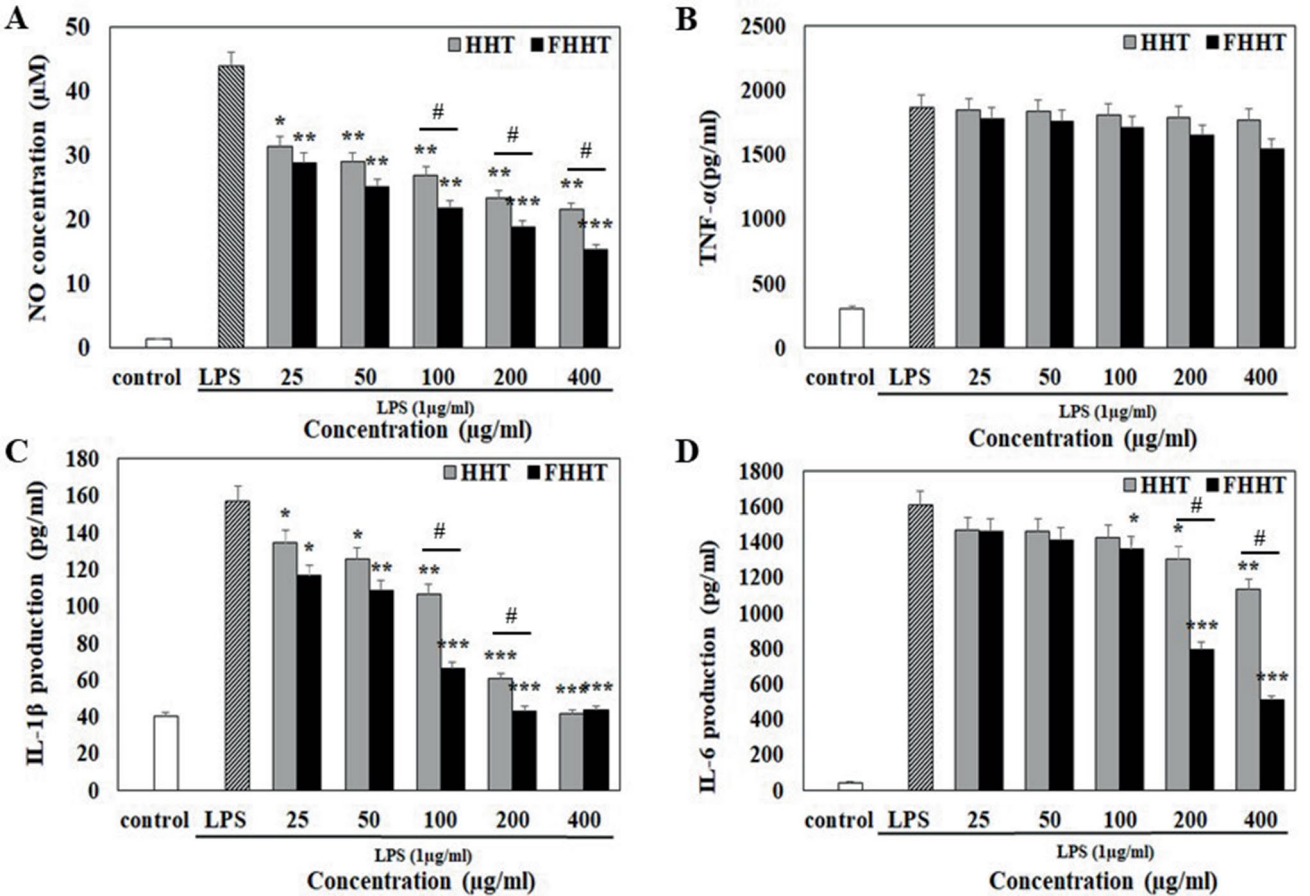
Anti-inflammatory activity of HHT and FHHT. The production of (**A**) NO, (**B**) TNF-α, (**C**) IL-1β, and (**D**) IL-6 levels were measured using Griess reaction (NO) or ELISA (TNF-α, IL-1β, and IL-6). The results are represented as mean ± SD from three independent experiments. * *p* < 0.05, ** *p* < 0.01, *** *p* < 0.001 indicates a significant difference w.r.t the LPS control. # *p* < 0.05 indicates a significant difference on HHT vs. FHHT. Note: HHT, Hwangryeonhaedok-tang; FHHT, fermented Hwangryeonhaedok-tang; LPS, lipopolysaccharide; PBS, phosphate-buffered saline; NO, nitric oxide; TNF, tumor necrosis factor; IL, interleukin; ELISA, enzyme-linked immunosorbent assay; SD, standard deviation

HHT and FHHT at concentrations of 25–400 µg/mL were used to estimate the anti-inflammatory effect of HHT and FHHT in RAW264.7 cells since they did not influence the viability of cells at this concentration (Fig. 5). NO is known to exhibit both pro- and anti-inflammatory effects depending on its concentration and physiological environment [45]. However, overproduction of NO in the host can induce inflammatory disorders such as sepsis leading to poor tissue perfusion, hypotension, and multiple organ failure [46]. In our study, HHT (25–400 µg/mL) and FHHT (25–400 µg/mL) significantly decreased cellular NO production compared to LPS-treated cells (Fig. 6A). In particular, FHHT tended to lower the NO level compared to that by HHT at all concentrations (25–400 µg/mL). To elucidate the factors responsible for the inhibition of NO production by HHT and FHHT treatments, we investigated the expression of pro-inflammatory cytokines such as tumor necrosis factor (TNF)-α, interleukin (IL)-1β, and IL-6. HHT and FHHT were seen to significantly decrease the production of IL-1β and IL-6 in LPS-treated cells (Fig. 6C and D). Notably, as seen in the effect on NO production, FHHT was more effective in lowering the production of pro-inflammatory cytokines compared to HHT. However, HHT and FHHT treatment had no significant effect on TNF-α production level from LPS-treated cells (Fig. 6B). Cytokines are produced from cells of the host immune system in response to injury or infection and act as mediators in immune and inflammatory reactions [47]. Pro-inflammatory cytokines such as TNF-α, IL-1β, and IL-6 affect the interaction between various intercellular, biochemical, and immunological mediators in inflammatory responses at multiple levels [48]. Thus, overexpression of these molecules can lead to pathological consequences in the host. Our findings demonstrate that HHT and FHHT exhibit anti-inflammatory activities that are enhanced by fermentation by attenuating NO production as a consequence of decreasing IL-1β and IL-6 levels.

## Conclusions

In the present study, HHT fermented with *Lpb. plantarum* Wikim0111 exhibited higher antioxidant and anti-inflammatory properties than unfermented HHT, as well as greater antibacterial activity against *C. acnes*. However, the control group was set without considering of the effects of YE fermented by *Lpb. plantarum* Wikim0111. Therefore, it is necessary to set up a control group that considers this point in a future study to conduct a comparative evaluation of the activities of the control group to those of HHT and FHHT. In addition, further studies in animal models and clinical settings are required. In conclusion, fermentation of HHT with *Lpb. plantarum* WiKim0111 significantly enhanced its antioxidant, anti-inflammatory, and antibacterial activities, particularly against *C. acnes*. These findings suggest that FHHT holds strong potential as a natural therapeutic agent for acne-related skin conditions, offering a promising complementary or alternative approach to conventional treatments, especially those associated with side effects or antibiotic resistance.

## Data Availability

The datasets used and analyzed during the current study are available from the corresponding author on reasonable request.
